# Repatriation of a historical North Atlantic right whale habitat during an era of rapid climate change

**DOI:** 10.1038/s41598-022-16200-8

**Published:** 2022-07-20

**Authors:** O. O’Brien, D. E. Pendleton, L. C. Ganley, K. R. McKenna, R. D. Kenney, E. Quintana-Rizzo, C. A. Mayo, S. D. Kraus, J. V. Redfern

**Affiliations:** 1 Anderson Cabot Center for Ocean Life at the New England Aquarium, Boston, MA 02110 USA; 2 Graduate School of Oceanography, University of Rhode Island, Narragansett, RI 02882 USA; 3 Department of Biology, Simmons University, Boston, MA 02115 USA; 4 Center for Coastal Studies, Provincetown, MA 02657 USA

**Keywords:** Ecology, Climate-change ecology, Conservation biology

## Abstract

Climate change is affecting species distributions in space and time. In the Gulf of Maine, one of the fastest-warming marine regions on Earth, rapid warming has caused prey-related changes in the distribution of the critically endangered North Atlantic right whale (*Eubalaena glacialis*). Concurrently, right whales have returned to historically important areas such as southern New England shelf waters, an area known to have been a whaling ground. We compared aerial survey data from two time periods (2013–2015; 2017–2019) to assess trends in right whale abundance in the region during winter and spring. Using distance sampling techniques, we chose a hazard rate key function to model right whale detections and used seasonal encounter rates to estimate abundance. The mean log of abundance increased by 1.40 annually between 2013 and 2019 (p = 0.004), and the mean number of individuals detected per year increased by 2.23 annually between 2013 and 2019 (R^2^ = 0.69, p = 0.001). These results demonstrate the current importance of this habitat and suggest that management options must continually evolve as right whales repatriate historical habitats and potentially expand to new habitats as they adapt to climate change.

## Introduction

Climate change is affecting the temporal and spatial distributions of marine species worldwide^1–4^. Warming temperatures may cause phenological changes, such as earlier or later arrivals in habitats^2^, longer residence times^3^, or a mismatch in important predator–prey processes^5–8^ (e.g., mismatches between migration timing and prey maturation). Changes in spatial distributions resulting from climate change include movements poleward, range expansion, range contraction, or changes in migratory behavior^9–12^.

The Gulf of Maine (GOM) in the Northwest Atlantic has warmed faster than ~ 98% of the ocean^13,14^, and warming is expected to continue^15^. The rapid pace of this warming has already affected many species including zooplankton^16^, commercially important fishes^17^, birds^18^, and baleen whales^19^. Of particular concern are changes caused by warming in the distribution of the critically endangered North Atlantic right whale (*Eubalaena glacialis;* hereafter, right whale). A bottom-water regime change occurred in the GOM between 2008 and 2010^16^ and this change coincided with the beginning of a decline in right whale numbers^20^ and a decrease in their birth rate^21^. The decrease in birth rates could be related to changes in the availability of one of the primary prey of right whales, *Calanus finmarchicus*, which has been negatively impacted by warming bottom temperatures^9,16,22,23^and other climate-associated changes (e.g., earlier spring transition dates^23^). In addition to these changes, there were major changes in right whale distribution^9,24^ and an increase in lethal entanglements in fishing gear and ship strikes^25,26^.

**Figure 1:**
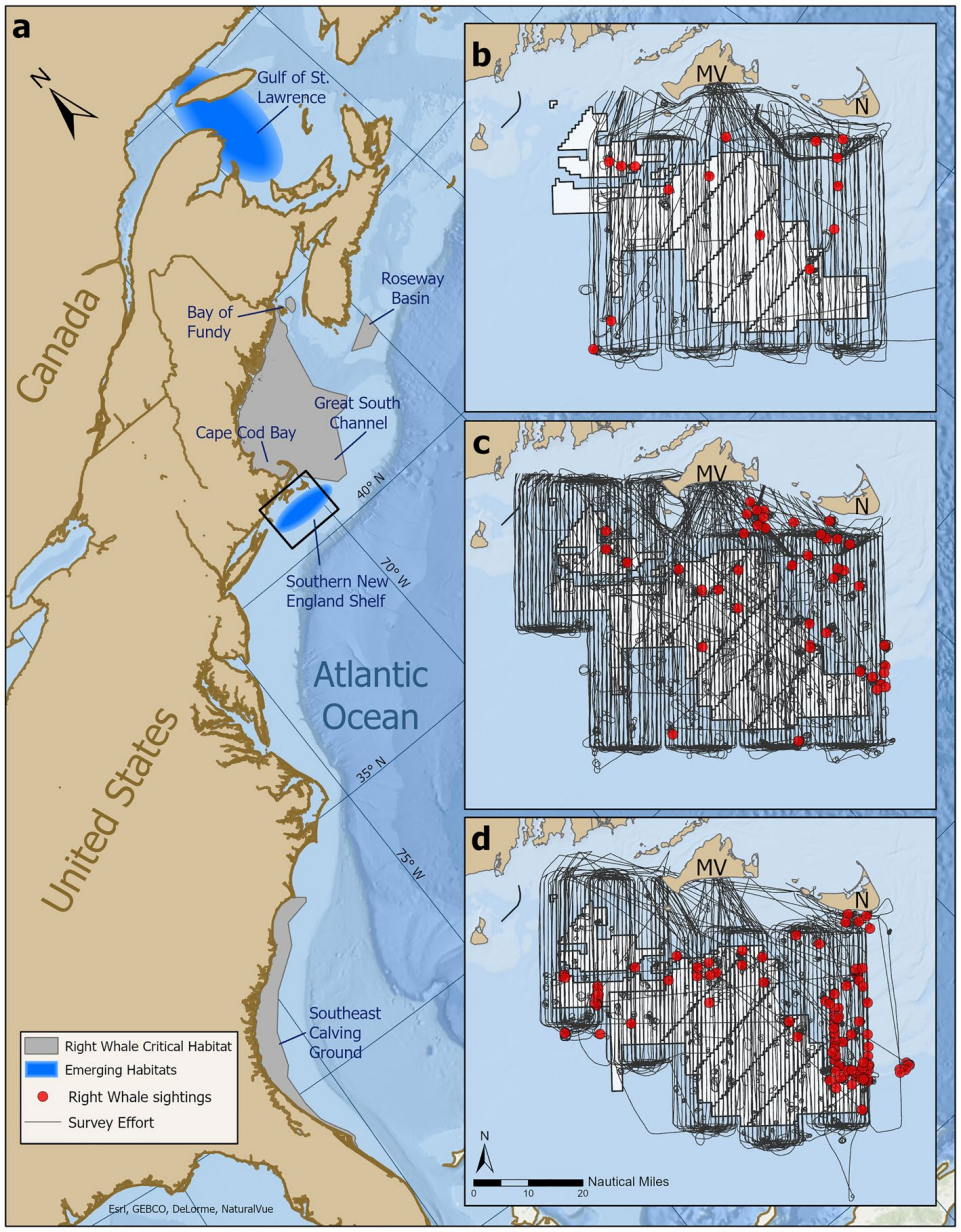
Known right whale habitats in the Northwest Atlantic. (**a**) Gray polygons encompass known right whale habitats; blue ovals represent emerging habitats. Black box and insets show the New England Aquarium broad-scale survey area. (**b**–**d**) Broad-scale survey effort (black lines) and right whale sightings (red circles) during three different time periods: (**b**) 2011–2012, (**c**) 2013–2015, (**d**) 2017–2019. White shading represents MA/RI wind energy lease areas. MV = Martha’s Vineyard, N = Nantucket. Figure was created using ArcGIS Pro (version 2.9.2).

Prior to 2010, right whales followed a reliable migration pattern that was driven by the distribution patterns of their prey^27,28^ (Fig. 1a). Right whales spent winter months (December–February) calving and socializing in coastal waters of the southeastern United States^27^ or feeding in Cape Cod Bay, Massachusetts^29–31^. Right whales were observed in the Great South Channel east of Cape Cod, Massachusetts, in the spring^32^ and the Bay of Fundy and Roseway Basin, in the Canadian Maritimes, in the summer^33^. Right whale distribution in the fall has not been well understood, but whales have been documented at Jeffreys Ledge^34^ (offshore of New Hampshire) and Jordan Basin (central GOM)^35^ during October and November.

Right whale occurrence decreased substantially or fell to zero in previously important feeding areas (i.e., Bay of Fundy^26^, the Great South Channel^9^) around 2010. In contrast, right whale abundance in Cape Cod Bay increased^36^. Right whales were also observed in large numbers in a new feeding area, the Gulf of St. Lawrence in Canada^26,37^, and in smaller numbers in a historically important area, the waters south of Nantucket and Martha’s Vineyard—the eastern portion of the southern New England shelf (SNE; Fig. 1a)^38,39^.

These changes in right whale distributions have complicated the conservation and management of the species. Right whale management strategies are well-established in historically important habitats and have included the creation of critical habitat and management of fishing gear, ship traffic patterns, and ship speeds^33,40–42^. Similar protections were not initially in place in the new right whale feeding area, the Gulf of Saint Lawrence, and the increase in right whale mortalities associated with changing distributions have largely occurred in this area^26^.

SNE is not a new habitat for right whales. Early whalers hunted right whales, and, likely, humpback (*Megaptera novaeangliae*) and sperm whales (*Physeter macrocephalus*), off Nantucket and Martha’s Vineyard in addition to other areas off New England (Rhode Island, Connecticut, and Long Island, New York). Shore-based whaling off Nantucket and Martha’s Vineyard began in the late 1600s^43,44^. By the early eighteenth century, whaling effort had likely moved farther offshore^43^. However, Allen^44^ noted that groups of right whales remained off Nantucket and the nearby smaller islands of Muskeget and Tuckernuck in March and April during the eighteenth century. Since the beginning of modern survey effort in the late 1970s, small numbers of right whales have been periodically documented in SNE^45,46^. Habitat suitability models developed using data collected between 2002 and 2006 predicted this area to be a potentially important right whale feeding habitat^47^. These predictions were unexpected because this area had been considered part of the right whale migration corridor rather than a feeding ground.

In 2013, SNE was designated for wind energy development by the Bureau of Ocean and Energy Management, and currently there are plans to develop the first U.S. large-scale commercial wind farm in this area. Kraus et al.^48^ hypothesized three impacts of wind farms on marine mammals: displacement, behavior disruption, and stress. However, no wind farm of this scale has been constructed in important large whale habitat. Consequently, it is critical to develop mitigation solutions to minimize any potential negative effects of construction, operation, and de-commissioning.

Systematic aerial surveys of marine megafauna in SNE began in late 2011 to collect the data needed to develop these mitigation measures. Surveys conducted between 2011 and 2015 documented small numbers of right whales in SNE during the winter and spring^38^. More recent surveys have suggested changes in right whale use of this area^49^, but trends in right whale abundance have not been assessed quantitatively. Assessing trends in abundance is challenging because the amount and type of survey effort has changed through time. We addressed this knowledge gap using data from aerial surveys to estimate seasonal right whale abundance. We assessed changes in right whale use of SNE by quantifying trends in seasonal abundance and in the number of individually identified right whales. Increasing trends would suggest increased importance of the SNE habitat to right whales, and thus an urgent need to effectively manage human activities in this area.

## Methods

### Study area and survey methods

The New England Aquarium began conducting systematic aerial surveys of SNE in October 2011. Broad-scale surveys were conducted approximately twice a month (weather dependent) from October 2011 to November 2012 in waters being designated for wind energy development off southern Massachusetts (total area surveyed was 6911 km^2^; Fig. 1b). At the time of survey design, this area was also predicted to be an area of favorable right whale habitat^46^. In December 2012, the survey area was expanded to include waters being designated for wind energy development off Rhode Island. This larger area (7789 km^2^) was surveyed through June 2015 (Fig. 1c). A gap in surveys occurred between July 2015 and January 2017. When broad-scale surveys resumed in February 2017, they were focused on a smaller area (5811 km^2^) surrounding wind energy lease zones (Fig. 1d). We used the area surveyed in the most recent years (i.e., 2017–2019) as our study area. We excluded the 2011 and 2012 data from our analyses (with the exception of December 2012) because surveys conducted in these years did not cover the entire study area.

Broad-scale surveys were designed to cover the entire study area in one day. Tracklines were either 13 (during 2011 to 2015) or 11 (during 2017 to 2019) km apart (7 or 6 nautical miles, nm, respectively). The start point of the survey was randomly drawn from nine (during 2011 to 2015) or eight (during 2017 to 2019) options. Each option shifted all tracklines 1.4 km (0.75 nm) east, but maintained the spacing between tracklines. During the study period, condensed surveys were also flown from 2017 to 2019. These surveys were designed to cover areas used by aggregations of right whales. Tracklines were 6 km apart (3 nm). Both survey types followed distance sampling protocols.

Surveys were conducted using a Cessna Skymaster O-2A, a twin-engine, high-wing aircraft. Surveys were flown at an altitude of 305 m (1000 ft) and a ground speed of approximately 185 km/h (100 kts). Surveys were typically flown in the following conditions: wind speed ≤ 19 km/h (10 kts), Beaufort sea state < 4, and visibility ≥ 9 km (5 nm). A computer data-logger system^50^ automatically recorded flight parameters (e.g., time, latitude, longitude, heading, altitude, speed) at frequent intervals (every 2 to 5 s). Two observers, one on each side of the aircraft, scanned out to 3.7 km (2 nm) for large whales, dolphins, and sea turtles; the observers could not see directly below the aircraft. Observers recorded the following environmental data: general weather conditions (clear, overcast, hazy, etc.), visibility, Beaufort sea state, cloud cover, and sun glare. When an animal was sighted, the sighting distance was recorded when the animal was abeam of the aircraft using distance bands marked on the aircraft’s wing struts. Surveys were conducted in closing mode: after observers marked the sighting distance, the aircraft diverted from trackline to photograph animals, obtain group size estimates, and confirm species identification. If a sighting was made during a diversion from the trackline, the location of the sighting was recorded and the distance to the trackline was measured during post-processing. Photographs of rostral callosity patterns were taken for all right whales when possible. Photographs were used to identify individuals by comparing the callosity patterns to those in the North Atlantic Right Whale Consortium Catalog^51^. Detailed quality-control of all sighting and effort data was conducted by the North Atlantic Right Whale Consortium when data were submitted for inclusion in their database.

### Abundance estimation

We used distance sampling methodology^52^ to estimate seasonal right whale abundance. To estimate effective strip width (i.e., the area effectively searched on each side of the aircraft), we fit a detection function to our sighting data using the R package ‘Distance’^53,54^. Fitting a detection function requires an adequate sample size, which is at least 25–30 sightings and ideally 60–80 sightings^55^. We did not have the required sample size to fit a detection function to each year of survey data. Consequently, we fit a detection function using right whale sightings from the entire study period (i.e., 2011–2019). Although previous studies^38^ have reported on right whale abundance for 2013–2015, those estimates used a detection function fit with all large whale sightings, and so we recalculate those years here. We used sightings collected during all survey effort because standard distance sampling protocols were followed during all surveys, although only broad-scale survey effort was used for abundance estimation. We left-truncated the data at 0.15 km because observers could not see directly below the aircraft. We visually assessed a histogram of sightings distances and determined that the data should be right-truncated at 1.8 km. Initial testing of environmental covariates (Beaufort sea state, glare, and visibility) showed that they did not affect detection distance so they were not included in final analyses. Hazard rate and half-normal models were used to evaluate detection probabilities as a function of perpendicular distance, and model fit was checked using a Chi-squared goodness-of-fit test.

Density for each trackline was calculated using the standard line-transect equation:

1 $$D=\frac{n \cdot s}{2\mu L}$$

where *n* is the number of sightings, *s* is the season-specific average group size, *L* is length of trackline surveyed on-effort, and µ is the effective strip width estimated by the pooled detection function (i.e., the distance at which as many animals are seen beyond µ as are missed within µ, derived using all years of survey data). The number of detections per unit distance ($\frac{n}{L}$) is also referred to as the encounter rate. Density estimates used sightings with a definite or probable species identification that were made under the following conditions: altitude ≤ 366 m, visibility ≥ 3.7 km, and Beaufort sea state ≤ 3. Density estimates do not include sightings made while transiting to the study area or during transits between tracklines. Density estimates used sightings from broad-scale surveys that were made on tracklines or when the aircraft diverted from the trackline to photograph animals, obtain group size estimates, and confirm species identification. Survey mileage from these diversions is not included in the effort calculations because the probability of detecting animals is reduced during these diversions (e.g., search effort is reduced when photographing animals).

Including sightings from trackline diversions is not standard however, aerial survey protocols would likely result in negative bias in right whale encounter rates and density estimates if we excluded sightings obtained during these events. In particular, the aircraft diverts from the trackline for almost all right whale sightings. For sightings made from the trackline or during these diversions, the aircraft typically stays with the whales until they dive. Consequently, the whales sighted during trackline diversions are not available for detection when the aircraft returns to the trackline. This potential for negative bias is likely higher in years with more sightings, which could affect the trend assessment.

The average density in the study area for each season was calculated using the effort-weighted mean density of all tracklines. We multiplied the estimated average density by the size of the study area (defined as the area surveyed during 2017 to 2019) to obtain seasonal abundance estimates. Seasons were defined as winter (December to February), spring (March to May), summer (June to August), and fall (September to November), e.g., winter 2013 includes December of 2012. The probability of detecting animals on the trackline, g(0), has not been estimated for our study area and was assumed to be one for our analyses (i.e., perfect trackline detection). Consequently, these estimates represent uncorrected density and abundance.

‘Distance’ calculates the variance of the density estimate additively from the variance of the component parameters: encounter rate, detection function, and group size. *Distance* provides upper and lower 95% confidence limits for the abundance estimates and calculates the coefficient of variation:

2 $$CV =\frac{\surd v}{a}$$

where *v* = variance and *a* is the mean abundance.

### Trends in right whale use of SNE

#### Trends in abundance

Right whales were not observed in the survey area during summer and fall from 2013 to 2015. Consequently, we only assessed trends in winter and spring right whale abundance. We fit generalized linear models (GLM) to assess abundance trends between 2013 and 2019, fitting the models in R using a logarithmic link and gamma error distribution^53^. Within each year, we used two abundance estimates (winter abundance and spring abundance). We used year and season as covariates to explain trends in abundance. Using the GLM, we determined that the season covariate was not significant. Therefore, we refit the model using year as the only covariate and we report the results of this model.

The variance in abundance estimates for species with small sample sizes is typically large^56^. However, Durant et al.^56^ found that distance data with small samples and large confidence intervals could be used to estimate density trends over long periods of time. We used the inverse of the squared coefficient of variation for each abundance estimate as weights in our model to incorporate this large source of uncertainty into the trend estimate.

#### Trends in the number of individual right whales

Detecting trends in abundance can be challenging because of the large variances associated with the abundance estimates. Therefore, we also assessed trends in relative abundance using the number of unique individual right whales observed each season per 1000 km of effort (IPUE). All on-effort data from broad-scale surveys were used in these analyses, including transits between tracklines, and over water transits to the survey area. This methodology is consistent with the encounter rate and sightings per unit effort calculations in previous analyses of surveys in this area^38,39^, and enabled us to include data from effort that was excluded from the abundance estimates. We used the number of unique individuals instead of encounter rate or sightings per unit effort. Because almost all sighted right whales were photographed, this method ensures that duplicate sightings were not used. We used linear models to assess IPUE trends between 2013 and 2019 following the same process as we used for the abundance trends (linear models provided adequate fit so GLMs were not explored). Specifically, we started with a model using year and season as covariates, but found that season was not significant; consequently, results from the model with only the year covariate are reported.

## Results

### Survey effort

From December 2012 through July 2019, 82 days of broad-scale aerial survey effort were conducted covering 42,515 km of on-effort trackline (Table 1). On these surveys, 370 right whales were sighted and 327 were matched to a cataloged individual, resulting in 216 unique individuals. Total seasonal trackline effort ranged between approximately 7240 km (fall) and 15,430 km (spring). Two seasons during these six years had no survey effort: fall 2015 and fall 2019. Yearly trackline effort ranged between 4583 km (in 2019) and 10,415 km (in 2014).

**Table 1:** Broad-scale aerial survey effort (km) in Southern New England shelf waters.

Year	Winter	Spring	Summer	Fall	Total
2013	806	2521	2977	2444	8748
2014	1497	2615	4018	2285	10,415
2015	1894	4280	959	0	7133
2017	531	3016	1698	1605	6850
2018	1404	1797	593	906	4700
2019	2181	1201	1201	0	4583
Total	8313	15,430	11,446	7240	42,515

### Abundance estimation

**Figure 2:**
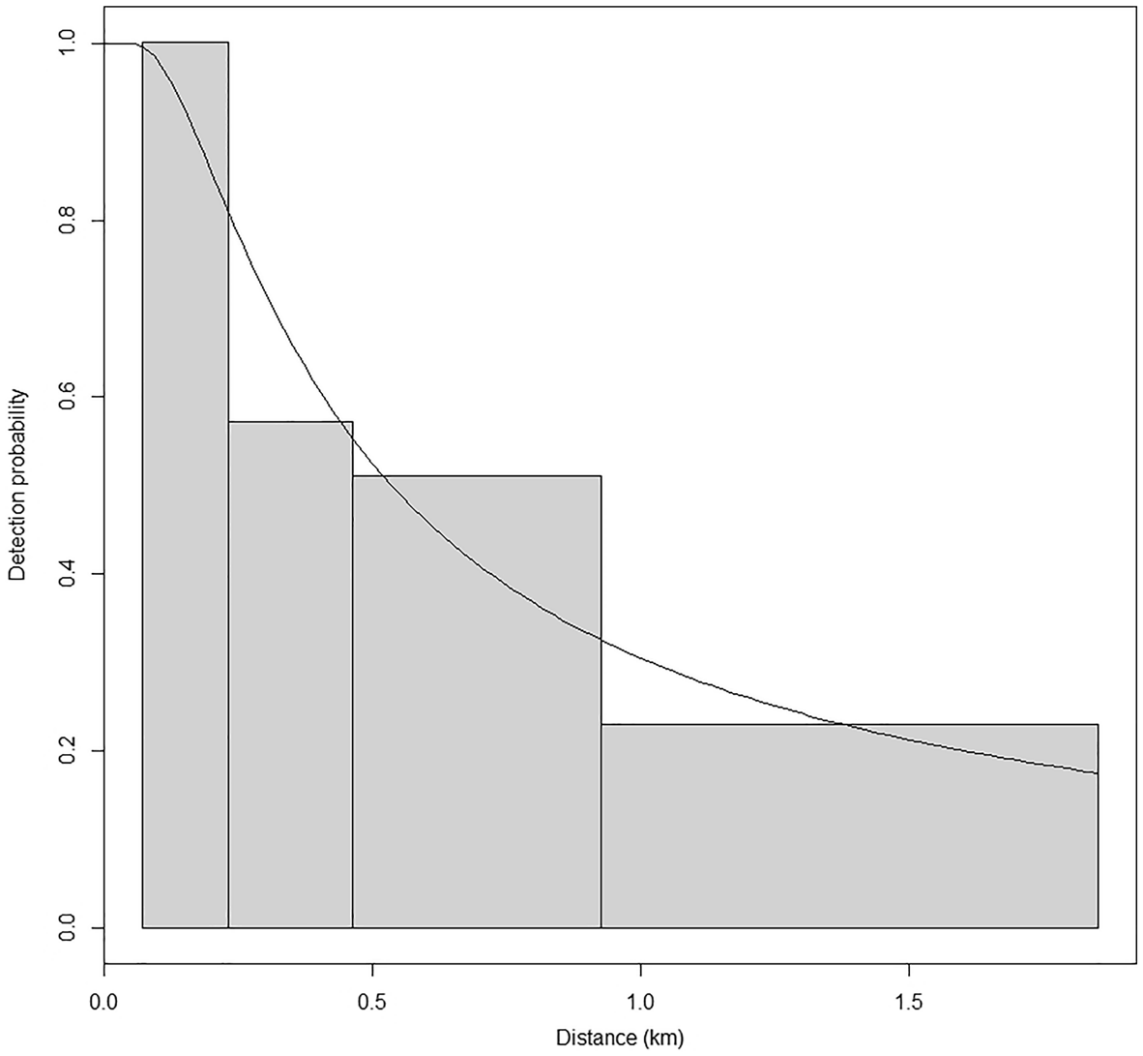
Right whale detection function derived from survey effort conducted between 2011 and 2019. The line represents the fitted detection function. Bars represent the frequency of sightings in predefined distance bins.

We selected a hazard rate detection function truncated at 1.8 km (Fig. 2). Truncating our data at 1.8 km resulted in the removal of 77 out of 234 total detections. This model estimated that the effective strip width was 0.72 km. We estimated abundance for each season starting in 2013 (Table 2). These estimates were not corrected for perception bias or for availability bias (i.e., we assume g(0) = 1) and represent a minimum number of whales present in each season. Abundance estimates for 2013–2015 were comparable to previously published estimates^38^. Seasonal abundance ranged from zero (summer and fall 2013, 2014, 2018; summer 2015) to 123 whales (winter 2019). Right whale winter abundances in 2017, 2018, and 2019 were higher than any winter abundance in 2013–2015, and we observed the same pattern in the 2017 and 2019 spring abundance estimates. Confidence intervals were wide, as is standard for estimates of large whale abundance. Encounter rates of rare animals can vary widely within an area and encounter rate is a large component of variance in line-transect surveys.

**Table 2:** Right whale seasonal abundance estimates (N), squared coefficients of variation (SCV), and 95% upper and lower confidence limits (95% CI). NE = no survey effort.

Year	Winter		Spring		Summer		Fall	
	N (SCV)	95% CI	N (SCV)	95% CI	N (SCV)	95% CI	N (SCV)	95% CI
2013	30 (0.35)	6.4–141.2	2 (0.10)	1.2–2.9	0	0	0	0
2014	11 (0.35)	1.2–123.2	15 (0.55)	3.5–55.2	0	0	0	0
2015	11 (0.35)	1.2–123.2	30 (0.26)	11.0–76.7	0	0	NE	NE
2017	53 (1.16)	8.1–363.2	48 (0.17)	21.5–104.0	2 (0.62)	0.6–9.9	5 (0.10)	2.9–9.3
2018	78 (0.16)	36.6–167.9	7 (0.39)	2.3–21.5	0	0	0	0
2019	123 (0.12)	62.8–241.7	81 (1.22)	8.1–764.7	30 (1.07)	5.2–186.5	NE	NE

Right whale IPUE ranged from zero (summer and fall 2013, 2014; summer 2015) to 19.05 whales per 1000 km in winter of 2019 (Table 3). Winter IPUE in 2017, 2018, and 2019 were higher than winter IPUE in 2014–2015. However, the IPUE in winter 2013 was the third highest in the time series. Right whale spring IPUE in 2017, 2018, and 2019 were higher than any spring IPUE in 2013–2015. All 2013–2015 summer and fall IPUE were zero and all 2017–2019 summer and fall IPUE were greater than zero (0.8–4.3 whales per 1000 km).

**Table 3:** Unique individuals per unit effort (IPUE, whales per 1000 km) identified on broad-scale surveys in Southern New England shelf waters. NE = no survey effort.

	IPUE			
Year	Winter	Spring	Summer	Fall
2013	12.49	0.24	0	0
2014	1.72	3.71	0	0
2015	4.30	3.80	0	NE
2017	5.99	7.49	3.62	0.78
2018	12.17	4.74	2.81	2.6
2019	19.05	14.03	4.26	NE

### Trends in right whale use of SNE

**Figure 3:**
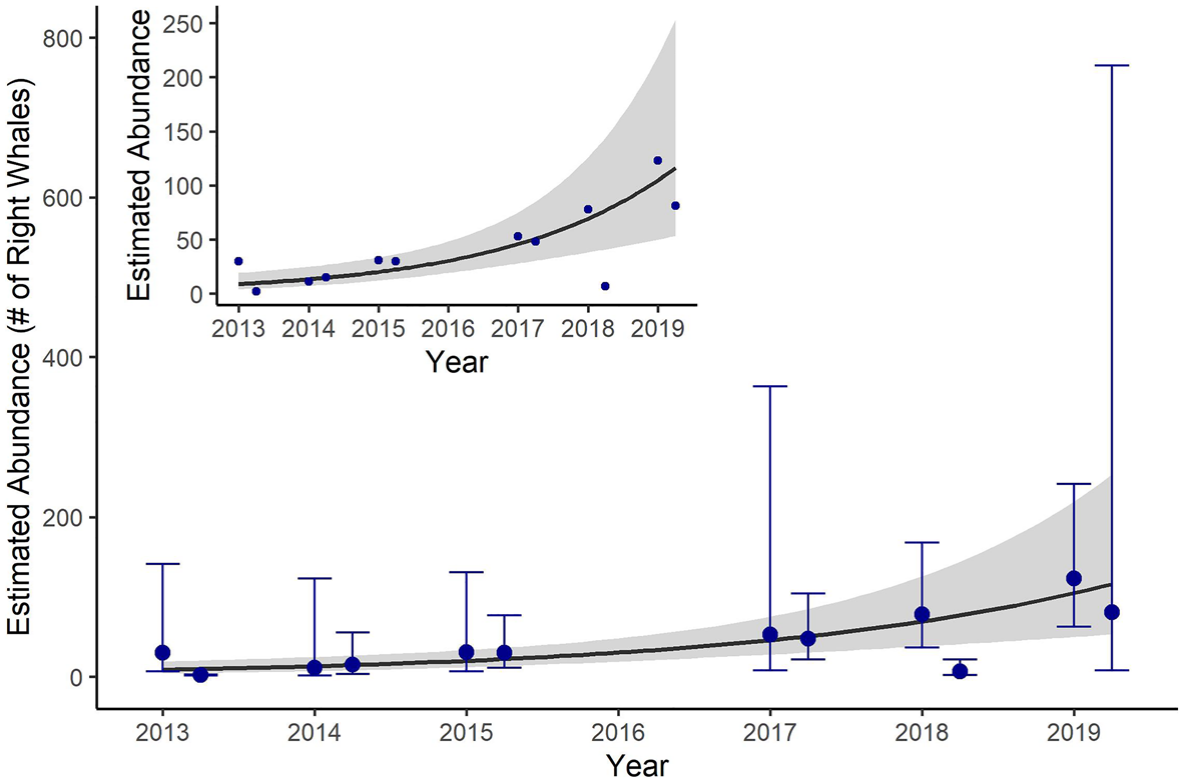
Trend in winter and spring right whale abundance estimated using a generalized linear model. Error bars represent 95% confidence intervals of abundance estimates; gray shading represents the 95% confidence interval around the trend line. Inset shows the same data and fit trend, without estimated abundance confidence intervals.

**Figure 4:**
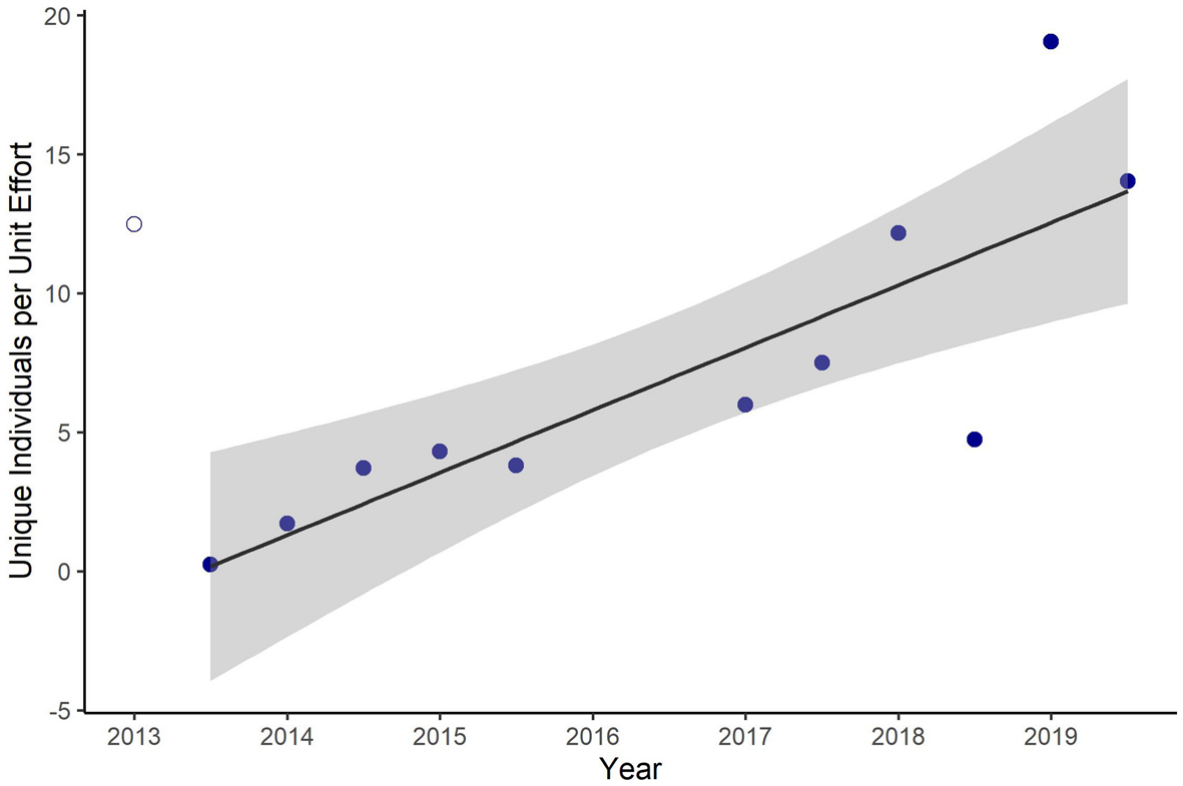
Trend in winter and spring unique individuals per unit effort estimated using a generalized linear model. Shading represents 95% confidence interval around the trend line. The open circle is an outlier that was removed from the analyses prior to fitting the trend line shown here (see text for explanation).

We found a significant, increasing trend in estimated right whale abundance between 2013 and 2019 in SNE (Fig. 3, β_year_ = 1.40, p = 0.004). We found a significant, increasing trend in right whale IPUE between 2013 and 2019 in SNE (β_year_ = 1.54, R^2^ = 0.31, p = 0.03). However, in the IPUE model, the Cook’s distance statistic indicated the winter 2013 season was an overly influential data point on the IPUE year coefficient (D = 1.00). Removal of this outlier did not affect the significance or direction of the IPUE annual trend (Fig. 4, β_year_ = 2.23, R^2^ = 0.69, p = 0.001).

Right whales were not detected during summer and fall from 2013 to 2015; however, right whales were detected during every season surveyed from 2017 to 2019. Abundance estimates were greater than zero in summer and fall 2017, and summer 2019 (Table 2). Right whale summer and fall abundances in 2018 were zero because all sightings occurred beyond the truncation distance or on transit legs. The 2019 summer abundance estimate was equal to or larger than all of the winter and spring abundance estimates during 2013–2015, although the confidence intervals for all estimates are large (Table 2). Similar patterns were observed in the seasonal IPUE. IPUE was zero for all summer and fall seasons in 2013–2015 but ranged from 0.78 to 4.26 whales per 1000 km in 2017–2019 (Table 3). The 2019 summer IPUE value was higher than any 2013–2015 spring value, although it was only higher than one of three 2013–2015 winter values (Table 3).

## Discussion

Our research documents right whale repatriation of a historically important habitat and contributes to our understanding of the recent changes in right whale distribution potentially caused by climate-driven habitat changes. Right whale occurrence in SNE has been documented since the whaling era (1600s–1900s), but right whale use of SNE has fluctuated. During the whaling era, SNE was likely an important right whale winter and spring habitat^43,44^. Shore-based whalers in Long Island, New York, were believed to primarily target migrating whales moving from winter calving grounds off the southeastern U.S. to feeding grounds in the GOM^57^. The general pattern of right whale occurrence in Massachusetts waters prior to our study was essentially identical to that described over a century ago by Allen^44^. Habitat models developed using data from 2002 to 2006 suggested that SNE contained right whale feeding habitat^47^; however, surveys conducted in the early 1980’s documented only small numbers of right whales in SNE^45^ and it was believed that this area represented part of a right whale migration corridor.

We found a significant, increasing trend in winter and spring right whale abundance in SNE from 2013 to 2019. Historically, right whale presence in SNE peaked in winter and spring^43,44^ and right whales were absent between June and October^43,44^. In our study, the historical seasonal occurrence of right whales in SNE appears to have continued through 2015. However, beginning in 2017, surveys began to detect small numbers of right whales in both summer and fall. The recent, year-round detection of right whales in SNE by aerial surveys is unique among major right whale habitats. Our analyses and previous studies^49,58^ suggest that SNE represents an increasingly important habitat for the declining right whale population.

Repatriation of historical whaling grounds is occurring around the world. Jackson et al. 2020^59^ documented the return of southern right whales (*Eubalaena australis*) and humpback whales to a whaling area off South Georgia Island in sub-Antarctic waters. The return of southern right and humpback whales to historic whaling areas has also been documented in South Australia, New Zealand, and the Coral Sea in the South Pacific^60–62^. In some areas, repatriation may not be possible because cultural memory of the habitat was lost or widespread whaling extirpated potential source populations^63^. For southern right whales and humpback whales, repatriation could represent range expansions as populations recover from whaling^59,60^, but this mechanism does not explain the repatriation of SNE by the declining North Atlantic right whale species^20^. For North Atlantic right whales, repatriation is likely driven by climate change.

Climate change has led to abundance and distribution changes in the right whale’s primary prey species, *C. finmarchicus*^29,64–66^. Right whales are found in areas that contain ultra-high prey concentrations relative to surrounding areas^9,29,31,47,67^. The repatriation of SNE by right whales is part of ongoing changes in their habitat-use patterns; in recent years, right whales have abandoned three previously important feeding habitats: the Bay of Fundy, the Great South Channel, and Roseway Basin^24,33,45,47^. In contrast, right whale habitat use has increased in Cape Cod Bay^36^ and the Gulf of St. Lawrence^37^. Our analyses indicate that SNE is another area where right whale habitat use has increased. The increased use of SNE could be a result of changes in prey within SNE or a decline in prey in other areas abandoned by right whales.

Our results and those of Quintana-Rizzo et al.^49^ demonstrate a novel summer occurrence of right whales in SNE from 2017 to 2019. The energetic tradeoffs of a long migration to other feeding grounds, such as the Gulf of St Lawrence, may increase the appeal of SNE to right whales during the summer months. To further assess the importance of this habitat, it would be valuable to understand the seasonal demographic patterns of the individuals using this area. The photographic identification data that are collected to assess demographic patterns could also be used to estimate abundance in a mark-recapture analysis. Using a mark-recapture analysis would allow the inclusion of a broader set of survey data (e.g., surveys in the study area conducted with a focus on obtaining photographic identification data) in the abundance estimation.

Sightings made during trackline diversions are not typically used to estimate density because including these sightings may positively bias the encounter rate and/or affect detection probability (detection probability can be positively or negatively biased). We included sightings made during trackline diversions in our analyses. To understand the potential bias caused by including these sightings, we compared our results to results from analyses that excluded these sightings. We found that there was still a significant and increasing trend in abundance, but the slope for the trend was smaller. The decrease in the slope is likely a result of excluding a large number of animals. Right whales tended to be aggregated; consequently, excluding sightings made during trackline diversions likely caused a negative bias in the abundance estimates and reduced the slope of the trend.

We also explored adding a group size covariate and seasonal, annual, and era (Early Era included years 2013–2015 and Late Era included years 2017–2019) covariates to our detection function because these covariates could affect our trend estimates. While we did not find that these covariates improved the detection function, the potential effects of these covariates should continue to be assessed as new data are collected. Finally, our density estimates assume that g(0) = 1 and do not account for animals missed while diving; consequently, they represent minimum densities and assume that g(0) has not changed during our study period. It would be useful to collect additional data to explore these assumptions.

In an era of climate-driven changes in species distributions, it is important to identify areas of novel habitat use and ensure species are protected in these areas. Our study shows that SNE has recently become a year-round right whale habitat and supports a substantial part of the species during winter and spring. Various fisheries use SNE and their fishing gear poses an entanglement risk to right whales^25,68^. Consequently, efforts to reduce entanglement risk in SNE are needed. Large-scale wind energy development is scheduled to begin in SNE in 2023, and will expose right whales to the short-term effects of construction, including noise from pile driving and increased vessel traffic^48^. Long-term impacts that may affect right whales in SNE include changes to vessel traffic and fishing patterns. It is acutely important to continue to monitor right whale abundance and distribution in this region to understand and mitigate the effects of wind energy development on this critically endangered species.
